# Are Veterans Getting Their Preferred Depression Treatment? A National Observational Study in the Veterans Health Administration

**DOI:** 10.1007/s11606-021-07136-2

**Published:** 2021-10-06

**Authors:** Lucinda B. Leung, Hannah N. Ziobrowski, Victor Puac-Polanco, Robert M. Bossarte, Corey Bryant, Janelle Keusch, Howard Liu, Wilfred R. Pigeon, David W. Oslin, Edward P. Post, Alan M. Zaslavsky, Jose R. Zubizarreta, Ronald C. Kessler

**Affiliations:** 1 Center for the Study of Healthcare Innovation, Implementation, and Policy, VA Greater Los Angeles Healthcare System, Los Angeles, CA USA; 2 Division of General Internal Medicine, and Health Services Research, UCLA David Geffen School of Medicine, Los Angeles, CA USA; 3 Department of Health Care Policy, Harvard Medical School, Boston, MA USA; 4 Department of Epidemiology, Columbia University Mailman School of Public Health, New York, NY USA; 5 Department of Behavioral Medicine and Psychiatry, West Virginia University, Morgantown, WV USA; 6 Center of Excellence for Suicide Prevention, Canandaigua VAMC, Canandaigua, NY USA; 7 VA Ann Arbor, Center for Clinical Management Research, Ann Arbor, MI USA; 8 Department of Psychiatry, University of Rochester Medical Center, Rochester, NY USA; 9 Cpl Michael J Crescenz VA Medical Center, VISN 4 Mental Illness Research Education and Clinical Center, Philadelphia, PA USA; 10 Perelman School of Medicine, University of Pennsylvania, Philadelphia, PA USA; 11 Department of Internal Medicine, University of Michigan Medical School, Ann Arbor, MI USA

**Keywords:** major depression, treatment preferences, treatment adherence, Veterans

## Abstract

**Background:**

Physician responsiveness to patient preferences for depression treatment may improve treatment adherence and clinical outcomes.

**Objective:**

To examine associations of patient treatment preferences with types of depression treatment received and treatment adherence among Veterans initiating depression treatment.

**Design:**

Patient self-report surveys at treatment initiation linked to medical records.

**Setting:**

Veterans Health Administration (VA) clinics nationally, 2018–2020.

**Participants:**

A total of 2582 patients (76.7% male, mean age 48.7 years, 62.3% Non-Hispanic White)

**Main Measures:**

Patient self-reported preferences for medication and psychotherapy on 0–10 self-anchoring visual analog scales (0=“completely unwilling”; 10=“completely willing”). Treatment receipt and adherence (refilling medications; attending 3+ psychotherapy sessions) over 3 months. Logistic regression models controlled for socio-demographics and geographic variables.

**Key Results:**

More patients reported strong preferences (10/10) for psychotherapy than medication (51.2% versus 36.7%, McNemar *χ*^2^_1_=175.3, *p*<0.001). A total of 32.1% of patients who preferred (7–10/10) medication and 21.8% who preferred psychotherapy did not receive these treatments. Patients who strongly preferred medication were substantially more likely to receive medication than those who had strong negative preferences (odds ratios [OR]=17.5; 95% confidence interval [CI]=12.5–24.5). Compared with patients who had strong negative psychotherapy preferences, those with strong psychotherapy preferences were about twice as likely to receive psychotherapy (OR=1.9; 95% CI=1.0–3.5). Patients who strongly preferred psychotherapy were more likely to adhere to psychotherapy than those with strong negative preferences (OR=3.3; 95% CI=1.4–7.4). Treatment preferences were not associated with medication or combined treatment adherence. Patients in primary care settings had lower odds of receiving (but not adhering to) psychotherapy than patients in specialty mental health settings. Depression severity was not associated with treatment receipt or adherence.

**Conclusions:**

Mismatches between treatment preferences and treatment type received were common and associated with worse treatment adherence for psychotherapy. Future research could examine ways to decrease mismatch between patient preferences and treatments received and potential effects on patient outcomes.

**Supplementary Information:**

The online version contains supplementary material available at 10.1007/s11606-021-07136-2.

## INTRODUCTION

Patient-centered care aims to be “respectful of and responsive to individual patient preferences, needs, and values.” This includes engaging in shared decision-making and patients receiving their preferred treatments unless clinically contra-indicated.^1,2^ Patient-centered care is associated with improved patient satisfaction, adherence, and clinical outcomes.^3–7^ While many determinants of treatment outcome are relatively immutable,^8–10^ certain aspects of patient-centered care, such as treatment based on patient preferences, can often be under clinician and health system control.

Depression, a common and disabling condition, can be effectively treated with two first-line options, of which most patients prefer psychotherapy over antidepressant medication.^11,12^ Although these preferences have little direct effect on comparative treatment response in controlled depression treatment trials,^13,14^ generalizability is limited to patients willing to be randomized to medication or psychotherapy. Mismatches between patient preferences and types of depression treatments received appear to play a more important role in the general population in predicting depression treatment initiation, adherence, and outcomes.^15,16^ Because medication and psychotherapy have roughly equivalent efficacy in treating mild–moderate depression,^17^ patients should be provided their preferred treatments when available and in the absence of severe disease, for which combined medication-psychotherapy is recommended.^18^ Treatment decisions in real-world settings are also influenced, though, by clinician judgments and other factors such as patient socio-demographic characteristics, comorbidities, disease severity, specialist capacity, and access to care. These factors may vary across treatment settings, making it unclear how often patients receive their preferred depression treatments.

The Veterans Health Administration (VA) offers a unique opportunity to examine the role of patient preferences on depression treatment receipt and adherence. The VA is the largest healthcare delivery system nationally that integrates mental health services into primary care.^19^ Veterans are heavily burdened by depressive disorders and are screened for depression annually in VA primary care settings.^20^ The VA then aims to provide ready access to treatment once depression is detected as part of high-priority national initiatives to improve mental health care and reduce Veteran suicides.^21^ Accordingly, Veterans seek mental health services preferentially within the VA even when they are dually eligible for care elsewhere.^22^ Clinical trials from over 15 years ago found that around a quarter of Veterans preferred only psychotherapy treatment for depression; however, results were mixed regarding whether matching Veteran preference improved patient outcomes (i.e., treatment adherence, depression symptoms).^23,24^ Outside of these clinical trials, no recent study has assessed depression treatment preferences among the VA patient population nationally. Based on these considerations, we examined whether treatment preferences predict types of depression treatment received and adherence to these treatments once received.

## METHODS

### Study Design and Cohort

Recruitment began with letters mailed to weekly nationally representative probability samples of eligible patients between December 2018 and June 2020. Eligibility for recruitment was defined as the patient having visited a VA outpatient clinic for treatment of major depression in the prior week and received an antidepressant medication prescription and/or a psychotherapy referral. Patients were excluded if they had any VA visit with a depression diagnosis, antidepressant prescription, or suicide attempt in the prior 365 days (as determined from electronic health records or patient self-reports). Exclusions were also made for lifetime diagnoses of autism, bipolar disorder, borderline intellectual functioning, dementia, intellectual disability, nonaffective psychosis, stereotyped movement disorders, and Tourette’s disorder and for prescriptions of antimanic or antipsychotic medications. (See Appendix Table 1 for ICD-9-CM/ICD-10-CM codes and Appendix Table 2 for medications.) During recruitment, we intentionally under-sampled patients that received medication, but not psychotherapy referral, during their first primary care–based visit to balance sample size across patient treatment groups. This was corrected by using a weight inversely proportional to the under-sampling prior to carrying out analysis.

Recruitment, detailed elsewhere,^25^ began with letters mailed to 55,106 potential study participants with an invitation to complete a 45-min self-report web- or phone-based baseline questionnaire with $50 incentives. Up to three recruitment calls were then made over 1 week, reaching 17,000 patients, 6298 of whom agreed to participate and 4164 completed the baseline questionnaire (24.4% response rate) (Appendix Figure 1). The Institutional Review Board of Syracuse VA Medical Center, Syracuse, New York, approved these procedures. We subsequently excluded 1526 baseline survey respondents because they either did not report depression as a presenting problem, reported a history of bipolar disorder or nonaffective psychosis, reported current suicidality, or reported subthreshold depression severity in the 2 weeks before baseline assessment. Twenty-eight respondents did not report treatment preferences. Analysis focused on the remaining 2582 respondents.

### Measures

#### Treatment Receipt and Adherence

Antidepressant medication receipt was defined as a prescription being picked up by or mailed to the patient. Adherence was defined as the patient having at least 60 days of any antidepressant during the first 84 days from the first prescription fill. Psychotherapy receipt was defined as a psychotherapy referral being completed for the patient. Adherence was defined as the patient attended 3+ psychotherapy sessions within 84 days of the referral. We used established methods for generating population-based metrics for adherence based on VA and National Committee for Quality Assurance (NCQA) guidelines.^26^

#### Treatment Preferences

Patients reported how willing they were to try medication and psychotherapy for their depression using a self-anchoring 0 (*unwilling*) to 10 (*completely willing*) visual analog scale (VAS) for each treatment type, which represented their treatment preferences.^27^ Responses were collapsed into categories labeled *strong positive* (10), *positive* (7–9), *neutral* (4–6), *negative* (1–3), and *strong negative* (0). A combined medication and psychotherapy preference rating was then created using the following hierarchical scheme: *strong positive* (if strong positive to both medication and psychotherapy), *positive* (if positive or strong positive to both), *neutral* (if no less than neutral to either), *negative* (if no less than negative to either), and *strong negative* (if strong negative to either or both).

#### Covariates

Several patient socio-demographic (age, sex, race/ethnicity, marital status, Census Block Group poverty level), geographic (country region, urbanicity), and clinical characteristics were abstracted from VA administrative data. Clinical characteristics included VA records for diagnoses of comorbid post-traumatic stress disorder (PTSD), substance use disorder (SUD), and scores on the revised Charlson Comorbidity Index^28^ categorized 0, 1, and 2+. Additional covariates were obtained from the baseline questionnaire. Depression symptom severity was assessed with the 16-item Quick Inventory of Depressive Symptomatology Self-Report Scale (QIDS-SR).^29^ Total scores were transformed into Hamilton Rating Scale for Depression (HRSD) severity levels of *none*, *mild*, *moderate*, *severe*, and *very severe*.^30^ Patients reported if their depressive episodes were brought on by some recent and/or long-term stressors or occurred “out of the blue.” Patients were also asked retrospectively about their exposure to childhood adversities based on the WHO World Mental Health Surveys^31^ and analyzed using latent class analysis to create multivariate maltreatment profiles. Based on the assumption that psychotherapy was less available in PC settings, we used administrative data to compare patients treated in primary care (PC) settings to those treated in specialty mental health (SMH) settings initially or within a month. Furthermore, we distinguished between PC clinics that had at least one full-time integrated mental health (PC-MHI) specialist on staff versus clinics without a full-time PC-MHI specialist.

### Analysis Methods

A multivariate weighting procedure was used to adjust for significant differences between baseline survey respondents and the full target sample using the R (3.6.1) program *sbw*.^32^ We used cross-tabulations applied to the weighted data to examine associations of patient preferences with treatment types received and then adherence. We compared the proportions of patients with strongly positive and strongly negative preferences for medications and psychotherapy using McNemar *χ*^2^ tests. Logistic regression analysis was then used to examine associations of patient preferences with types of treatment received and adherence controlling for socio-demographic and geographic variables, clinical features (depression severity, life stressors, comorbidity, childhood adversities), and treatment setting. We examined interactive effects between patient preferences and pertinent covariates (e.g., disease severity, treatment setting). Logistic regression coefficients were exponentiated to create odds ratios (ORs) and reported with 95% CIs. Statistical significance was consistently evaluated using .05-level two-sided tests. We adjusted for the false discovery rate using the Benjamini-Yekutieli method.^33^ Analyses were carried out using SAS 9.4.^34^

## RESULTS

### Sample Characteristics

The sample included 76.7% (*n*=1968) men and 23.3% (*n*=614) women. Most patients (62.3%, *n*=1712) were Non-Hispanic White. Mean (SD) baseline age was 48.7 years (15.3) (Table 1). More details on sample composition are reported elsewhere.^25^

**Table 1:** Sample Characteristics Compared to the Population from Which the Sample Was Recruited on a Range of VA Administrative Variables

	Sample (*n*=2582)	Population (*N*=55,106)	
	*n* (%)	*n* (%)	*χ*^2^
Age			32.2*
18–34	602 (23.5)	15564 (27.6)	21.3^†^
35–49	760 (29.4)	15038 (26.9)	7.5^†^
50–59	527 (19.8)	9229 (17.1)	12.6^†^
60+	693 (27.4)	15275 (28.4)	1.2
Male	1968 (76.7)	45276 (82.7)	61.0*
Race/ethnicity			154.4*
Non-Hispanic white	1712 (62.3)	34164 (60.8)	2.1
Non-Hispanic black	417 (18.7)	13344 (25.1)	54.8^†^
Hispanic	267 (11.0)	5530 (10.3)	1.4
Other	186 (8.0)	2068 (3.7)	116.3^†^
Marital status			66.8*
Currently married	1371 (52.5)	27176 (48.6)	15.4^†^
Previously married	810 (31.6)	15566 (28.7)	10.1^†^
Never married	401 (15.8)	12364 (22.7)	66.6^†^
Census region			2.8
Northeast	273 (10.6)	5685 (10.8)	0.1
Midwest	517 (19.0)	9998 (17.7)	2.8
South	1279 (50.0)	28727 (50.6)	0.4
West	513 (20.5)	10696 (20.9)	0.3
Urbanicity			0.6
Major metro	2152 (85.3)	46435 (85.9)	0.6
Urban	389 (13.3)	7800 (12.7)	0.6
Rural	41 (1.4)	871 (1.4)	0.0
% of population below 150% of poverty			24.4*
1st quartile	532 (21.2)	13643 (25.0)	19.6^†^
2nd quartile	657 (24.5)	14108 (25.0)	0.3
3rd quartile	697 (26.7)	13905 (25.0)	3.8
4th quartile	696 (27.6)	13450 (25.0)	8.8^†^

*Significant at the .05 level, two-sided test

†Individually significant at the .05 level, two-sided test, correcting false discovery rate

Data are weighted, but reported *n*’s are unweighted

### Associations of Treatment Preferences with Treatment Types Received and Adherence

More patients reported strong positive preferences (10/10) for psychotherapy than medication (51.2% vs. 36.7%; *χ*^2^_1_=175.3, *p*<0.001) and strong negative preferences (0/10) for medication than psychotherapy (16.0% vs. 2.0%; *χ*^2^_1_=15.3, *p*<0.001) (Table 2). A total of 25.1% of patients reported strong positive preferences for both psychotherapy and medication, 43.5% reported strong positive or positive preferences for both, 17.3% reported strong negative preference for at least one, and 35.0% reported either negative or strong negative preferences for both. Consistent with these preferences, more patients received a psychotherapy referral than a medication prescription (76.8% vs. 50.0%; *χ*^2^_1_=777.2, *p*<0.001), and 26.9% received both a psychotherapy referral and a medication prescription. A mismatch between preference and treatment received occurred for 23.7% of patients regarding medication (16.6% had positive preferences but did not receive it; 7.1% had negative preferences but received it) and 21.6% regarding psychotherapy (16.7% had positive preferences but did not receive it; 4.9% had negative preferences but received it). Treatment adherence was higher for psychotherapy than medication (44.7% vs. 36.1%; *χ*^2^_1_=23.6, *p*<0.001) and lowest for combined treatment (17.6%).

**Table 2:** Associations of Patient Treatment Preferences with Treatments Received and Adherence

	Preference distribution (*n*=2582)	Treatment received (*n*=2582)		Treatment adherence (*n*=1608 for medication, *n*=1774 for psychotherapy, *n*=800 for combined)	
Preference	*n* (%)	*n* (%)	OR (95% CI)	*n* (%)	OR (95% CI)
I. Medication					
Strong positive	1065 (36.7)	834 (68.9)	17.5* (12.5, 24.5)	353 (41.0)	1.8 (0.9, 3.4)
Positive	413 (14.9)	312 (65.4)	14.9* (10.3, 21.7)	102 (31.4)	1.2 (0.6, 2.4)
Neutral	430 (17.0)	251 (46.5)	6.9* (4.8, 9.9)	85 (34.3)	1.3 (0.6, 2.6)
Negative	353 (15.4)	147 (34.3)	4.3* (3.0, 6.2)	42 (27.0)	0.9 (0.4, 1.9)
Strong negative	321 (16.0)	54 (11.2)	1.0 (Ref)	17 (29.3)	1.0 (Ref)
Total	2582 (100.0)	1608 (50.0)	-	599 (36.1)	-
*χ*^2^_4_			357.3*		15.8*
II. Psychotherapy					
Strong positive	1312 (51.2)	936 (78.7)	1.9* (1.0, 3.5)	466 (50.3)	3.2* (1.4, 7.3)
Positive	657 (25.5)	454 (77.1)	1.7 (0.9, 3.2)	204 (44.6)	2.6* (1.1, 5.9)
Neutral	419 (16.2)	272 (73.8)	1.4 (0.8, 2.7)	92 (33.8)	1.7 (0.7, 3.9)
Negative	136 (5.1)	81 (70.4)	1.2 (0.6, 2.5)	21 (26.1)	1.2 (0.5, 3.0)
Strong negative	58 (2.0)	31 (66.1)	1.0 (Ref)	8 (22.9)	1.0 (Ref)
Total	2582 (100.0)	1774 (76.8)	-	791 (44.7)	-
*χ*^2^_4_			10.8*		41.3*
III. Combined					
Strong positive	710 (25.1)	293 (38.8)	7.5* (5.1, 11.1)	70 (24.3)	3.1 (0.9, 10.2)
Positive	516 (18.4)	197 (35.0)	6.4* (4.3, 9.5)	31 (13.3)	1.5 (0.4, 5.0)
Neutral	570 (21.5)	177 (27.3)	4.6* (3.1, 6.8)	25 (15.2)	1.6 (0.5, 5.4)
Negative	425 (17.7)	96 (19.7)	3.1* (2.0, 4.7)	12 (13.6)	1.3 (0.4, 4.9)
Strong negative	361 (17.3)	37 (7.7)	1.0 (Ref)	5 (10.2)	1.0 (Ref)
Total	2582 (100.0)	800 (26.9)	-	143 (17.6)	-
*χ*^2^_4_			128.1*		13.5*

*Significant at the .05 level, two-sided test

Based on multivariate models controlling for socio-demographics and geographic variables

Data are weighted, but reported *n*’s are unweighted

Patient treatment preferences were associated with types of treatment received for medication, psychotherapy, and combined treatment in models that controlled for socio-demographics and geographic variables (Table 2). Relative odds of receiving treatment based on increasingly positive preferences (compared to strong negative preferences) were very strong for medication, with ORs ranging from 4.3 (95% CI=3.0–6.0) for negative to 17.5 (95% CI=12.5–24.5) for strong positive preferences. For psychotherapy, relative odds of receiving this treatment based on increasingly positive preferences (compared to strong negative preferences) were weaker and only significant for those who strongly preferred psychotherapy (OR=1.9; 95% CI=1.0–3.5). The associations of treatment preference with receiving combined treatment were also strong. Compared with individuals with strong negative preferences for combined treatment, ORs ranged from 3.1 (95% CI=2.0–4.7) for negative to 7.5 (95% CI=5.1–11.1) for strong positive preferences.

Associations between patient preferences and treatment adherence were considerably weaker than for treatment receipt, but with a wider range of ORs for psychotherapy (from 1.2 [95% CI=0.5–3.0] for negative to 3.2 [95% CI=1.4–7.3] for strong positive preferences) than medication (0.9 [95% CI=0.4–1.9] for negative to 1.8 [95% CI=0.9–3.4] for strong positive preferences). For combined treatment adherence, ORs ranged from 1.3 for negative to 3.1 for strong positive preferences, but all 95% CIs crossed 1.0.

### Associations of Depression Severity with Treatment Preferences

Depression severity was associated with preferences for medication (*χ*^2^_12_=34.1, *p*<0.001), psychotherapy (*χ*^2^_12_=25.8, *p*=0.012), and combined treatment (*χ*^2^_12_=34.8, *p*<0.001) in models that controlled for socio-demographics and geographic variables (Appendix Table 3). Compared to patients with mild depression, patients with more severe depression were generally more likely to have neutral, positive, or strongly positive preferences for each type of treatment than strong negative preferences. But these associations were relatively weak in substantive terms, as indicated by the proportion of patients holding strong positive preferences increasing only modestly across the range of the depression severity scale for medication (34.3–40.9%), psychotherapy (45.8–54.4%), and combined treatment (21.4–29.2%).

### Associations of Depression Severity and Treatment Preferences with Treatment Setting

Compared with patients with mild depression, those with severe depression, but not very severe depression, had slightly greater relative odds of receiving treatment in a SMH setting (OR=1.4; 95% CI=1.1–1.8) (Appendix Table 4). Treatment setting was dichotomized for this analysis (i.e., PC vs. SMH) due to the small number of respondents treated in a PC setting where there was no full-time PC-MHI specialist. Treatment preferences were not associated with treatment setting. Interactions between depression severity and treatment preferences were nonsignificant in predicting treatment setting.

### Joint Associations of Treatment Preferences, Depression Severity, and Setting with Treatment Received and Adherence

We extended our models in Table 2 by additionally controlling for depression severity, treatment setting, clinical severity, and childhood adversity (Appendix Tables 5 and 6). Associations of treatment preference with medication receipt were relatively unchanged and remained strong in magnitude (Appendix Table 5). We found that the associations of treatment preferences with combined treatment receipt (Table 2) were due to patients with positive preferences for medication having higher relative odds of receiving combined treatment (OR=1.8; 95% CI=1.4–24) and those with negative preferences having lower relative odds of receiving combined treatment (OR=0.5; 95% CI=0.4–0.7), but not due to psychotherapy treatment preferences. Preferences had no significant interactions with depression severity, treatment setting, or the control variables in predicting treatment types received. In these models, depression severity was not associated with treatment type received, while treatment setting was strongly associated with receiving psychotherapy and combined treatment but not medication.

Associations of treatment preferences with psychotherapy and medication adherence were also essentially the same after additional adjustment (with estimates in Table 2 changing by no more than 0.1) (Appendix Table 6). Preferences neither for medication nor for psychotherapy predicted combined treatment adherence. No significant interactions were found of preferences with any of the control variables in predicting adherence to these treatments. In these models, neither depression severity nor treatment setting was associated with medication, psychotherapy, or combined treatment adherence.

## DISCUSSION

This is one of the largest studies to examine associations of patient treatment preferences with types of depression treatment received and adherence to those treatments.^35^ Consistent with prior studies,^36–38^ patients reported higher preferences for psychotherapy than medication. However, unlike studies in other primary care settings,^39^ a higher proportion of patients were referred to psychotherapy (76.8%) than prescribed medication (50.0%), presumably reflecting VA’s substantial investment in embedding integrated mental health specialists in primary care clinics. There was no evidence of an association between preference for psychotherapy and receipt of psychotherapy, whereas preference for medication was very strongly associated with receiving medication. The associations of preference with treatment adherence, in comparison, were stronger for psychotherapy than medication. Psychotherapy patients reporting a preference for psychotherapy in the baseline survey were twice as likely to be adherent over the next 3 months as patients with baseline negative psychotherapy preference. Furthermore, our study found that patients do not necessarily strongly adhere to, nor do they prefer, combined psychotherapy and medication treatment, which is often idealized by clinicians and experts. Future research could examine different ways to decrease mismatch between patient preferences and treatments received and potential effects on patient outcomes. For example, one may explore effects of increasing access to new virtual treatment options or of increasing fidelity to patient-centered medical home models that support shared decision-making on increasing treatment adherence, and subsequent treatment outcomes.

While we found no strong predictor of receiving medication other than patient preference, receiving psychotherapy was strongly predicted by treatment setting. The fact that this association was less pronounced in primary care settings that had full-time (OR=0.5) than part-time (OR=0.2) embedded PC-MHI specialists suggests that resource availability accounted for a meaningful part of the lower receipt of psychotherapy in PC settings.^40^ However, other researchers showed that clinician preferences are also involved.^38^ Indirectly to this point, no interactions were found between patient preferences and treatment setting. Barriers to receiving psychotherapy at the patient level might also be involved, such as inconvenience.^41^ Barriers to medication receipt need to be considered as well as clinical contra-indications, which were not considered here. Despite combined treatment being recommended for severe depression,^22^ depression severity was not observed to be associated with treatments received, including combined treatment.

To our knowledge, this study is among the first to assess Veteran preferences for depression treatment nationally but has several limitations. First, patient treatment preferences are complex and may not have been fully captured in the scales used here even though similar visual analog scales have been used in prior research on treatment preferences.^27,42^ Second, preferences were assessed an average of 1 week after initiating treatment. Patient preferences prior to that time might have been somewhat different. We also did not assess patients who initiated treatment other than psychotherapy or medication, as this study emphasized only guideline-concordant options. Third, the survey response rate was low (24.4%), although similar to rates reported in other studies.^43,44^ Respondents might have differed from non-respondents in important unmeasured ways, such as being more likely to try and adhere to depression treatment.^25^ Fourth, we did not account for prior treatment history, as the success or failure of prior treatment may influence patient preference and clinician judgment in depression treatment. Fifth, the study could not control for quality differences across similarly coded psychotherapy events. Finally, this study did not adjust for COVID-19-related disruptions in care, but 92.4% of study patients completed assessments before March 2020.

Within the context of these limitations, a much stronger association was found between patient preferences receiving preferred treatment for medication than psychotherapy. Psychotherapy preference, however, was a more important predictor of psychotherapy adherence than medication preference was of medication adherence. Substantial and comparable levels of mismatch were found between preferences and treatment received for medication (23.7%) and psychotherapy (21.6%). Contrary to treatment guidelines, no significant association was found between depression severity and combined treatment either alone or in interaction with preferences. This may reflect conservative practice styles among clinicians who prefer to combine treatment only if patients do not show improvement with a single treatment option. Nonetheless, mismatches between negative preferences and receipt of nonpreferred treatments do not appear to be selectively due to efforts on the part of clinicians to provide combined treatments to severe cases, pointing to the need for more patient-centered care.

The VA has invested heavily in national initiatives to increase reach of mental health services and appears to be providing largely accessible and mostly equitable care. Given the pervasiveness and disability associated with depression, future work should continue to explore where and for whom patient preferences are not being met when doing so is not clinically contra-indicated and how the match between preferences and treatments received can be increased in the service of improving treatment adherence and outcomes.

## Supplementary Information

ESM 1 (DOCX 77 kb)
