# Supplementary material for: Are Veterans Getting Their Preferred Depression Treatment? A National Observational Study in the Veterans Health Administration
Source: J Gen Intern Med. 2021 Oct 6;37(13):3235–41. doi: 10.1007/s11606-021-07136-2 (PMC8493943; doi:10.1007/s11606-021-07136-2)
Supplement: Supplementary file 1 — (DOCX 77 kb) [file 11606_2021_7136_MOESM1_ESM.docx]

**Supplementary Appendix**

| **Appendix Table 1. ICD-9-CM^1^ and ICD-10-CM^2^ Mental and Behavioral Health Codes for Major Depression and for exclusionary diagnoses** | |
| --- | --- |
|  |  |
| **Disorder** | **Codes** |
| Major depression | ICD-9-CM codes: 296.2X, 296.3X, 300.4, 311; ICD-10-CM codes: F32.XX (excluding F32.81 and F32.89), F33.XX |
|  |  |
| Bipolar disorder | ICD-9-CM codes: 296.0X, 296.1X, 296.4X, 296.5X, 296.6X, 296.7, 296.80, 296.81, 296.89, 301.13; ICD-10-CM codes: F30.X, F31.X, F34.0 |
|  |  |
| Nonaffective psychosis | ICD-9-CM codes: 293.81, 293.82, 293.89, 295.XX, 297.X, 298.X, 301.22; ICD-10-CM codes: F06.0, F06.1, F20.XX, F21, F22, F23, F24, F25.X, F28, F29, F53 |
|  |  |
| Dementia | ICD-9-CM codes: 290.XX, 294.1X, 294.8; ICD-10-CM codes: F01.XX, F01.XX, F03.XX |
|  |  |
| Intellectual disabilities | ICD-9-CM codes: 317, 318, 319; ICD-10-CM codes: F70, F71, F72, F73, F78, F79 |
|  |  |
| Autism | ICD-9-CM code: 299.XX; ICD-10-CM code: F84.0 |
|  |  |
| Tourette’s disorder | ICD-9-CM code: 307.23; ICD-10-CM code: F95.2 |
|  |  |
| Stereotyped movement disorders | ICD-9-CM code: 307.3; ICD-10-CM code: F98.4 |
|  |  |
| Borderline intellectual functioning | ICD-10-CM code: R41.83 |
|  |  |

Abbreviations. ICD-9-CM, International Classification of Diseases, Ninth Revision, Clinical Modification; ICD-10-CM, International Classification of Diseases, Tenth Revision, Clinical Modification.

| **Appendix Table 2. Exclusionary medications** | |
| --- | --- |
|  |  |
| **VHA Drug Classification** | **Medication Name** |
| Anticonvulsants | Brivaracetam, Cannabidiol, Carbamazepine, Cenobamate, Clobazam, Divalproex, Eslicarbazepine, Ethosuximide, Ethotoin, Felbamate, Fosphenytoin, Gabapentin, Lacosamide, Lamotrigine, Levetiracetam, Methsuximide, Oxcarbazepine, Perampanel, Phenytoin, Primidone, Rufinamide, Stiripentol, Tiagabine, Topiramate, Valproate sodium, Valproic acid, Vigabatrin, Zonisamide |
|  |  |
| Phenothiazine/ related antipsychotics | Chlorpromazine, Fluphenazine, Perphenazine, Thioridazine, Thiothixene, Trifluoperazine |
|  |  |
| Antipsychotics, other | Aripiprazole, Asenapine, Brexpiprazole, Cariprazine, Clozapine (Aurobindo), Clozapine (Caraco), Clozapine (Cozaril), Clozapine (Golden State), Clozapine (Mayne), Clozapine (Mylan), Clozapine (Sandoz), Clozapine (Teva), Clozapine (Versacloz), Haloperidol, Iloperidone, Loxapine, Lumateperone, Lurasidone, Molindone, Olanzapine, Paliperidone, Pimavanserin, Quetiapine, Risperidone, Ziprasidone |
|  |  |
| Lithium salts | Lithium |
|  |  |

| **Appendix Table 3. Associations of depression severity**^†^ **with treatment preferences (n=2,582)**^‡^ | | | | | | | | | | | | | | |
| --- | --- | --- | --- | --- | --- | --- | --- | --- | --- | --- | --- | --- | --- | --- |
|  | | | | | | | | | | | | | | |
|  | **Strong positive** | |  | **Positive** | |  | **Neutral** | |  | **Negative** | |  | **Strong Negative** |  |
| **Preference** | **n (%)** | **OR (95% CI)** |  | **n (%)** | **OR (95% CI)** |  | **n (%)** | **OR (95% CI)** |  | **n (%)** | **OR (95% CI)** |  | **n (%)** | **χ^2^_12_** |
| I. Medication |  |  |  |  |  |  |  |  |  |  |  |  |  | 34.1* |
| Very severe depression | 184 (40.9) | 1.9* (1.3, 2.9) |  | 66 (15.4) | 1.8* (1.1, 3.0) |  | 73 (18.4) | 2.0* (1.2, 3.2) |  | 57 (15.2) | 1.4 (0.9, 3.0) |  | 33 (10.1) |  |
| Severe depression | 223 (37.7) | 1.3 (0.9, 1.9) |  | 79 (14.8) | 1.3 (0.9, 2.0) |  | 112 (21.3) | 1.7* (1.1, 2.5) |  | 56 (11.8) | 0.8 (0.5, 1.2) |  | 59 (14.3) |  |
| Moderate depression | 345 (36.2) | 1.0 (0.8, 1.4) |  | 149 (16.2) | 1.2 (0.8, 1.7) |  | 128 (15.0) | 1.0 (0.7, 1.3) |  | 112 (14.5) | 0.8 (0.5, 1.1) |  | 123 (18.0) |  |
| Mild depression | 313 (34.3) | 1.0 (Ref) |  | 119 (13.4) | 1.0 (Ref) |  | 117 (15.5) | 1.0 (Ref) |  | 128 (18.9) | 1.0 (Ref) |  | 106 (18.0) |  |
| χ^2^_3_ | 12.0* | |  | 6.1 | |  | 17.0* | |  | 8.1* | |  |  |  |
| II. Psychotherapy preference |  |  |  |  |  |  |  |  |  |  |  |  |  | 25.8* |
| Very severe depression | 224 (54.4) | 0.7 (0.3, 1.6) |  | 104 (25.1) | 0.5 (0.2, 1.3) |  | 67 (16.4) | 0.6 (0.2, 1.3) |  | 9 (1.7) | 0.2* (0.0, 0.5) |  | 9 (2.5) |  |
| Severe depression | 287 (55.0) | 0.7 (0.3, 1.6) |  | 120 (23.0) | 0.5 (0.2, 1.1) |  | 79 (14.8) | 0.5 (0.2, 1.1) |  | 27 (4.6) | 0.4 (0.2, 1.1) |  | 16 (2.6) |  |
| Moderate depression | 443 (52.1) | 1.2 (0.5, 2.5) |  | 217 (25.6) | 1.0 (0.5, 2.1) |  | 128 (14.8) | 0.9 (0.4, 1.9) |  | 51 (5.8) | 1.0 (0.4, 2.3) |  | 18 (1.6) |  |
| Mild depression | 358 (45.8) | 1.0 (Ref) |  | 216 (27.5) | 1.0 (Ref) |  | 145 (18.6) | 1.0 (Ref) |  | 49 (6.4) | 1.0 (Ref) |  | 15 (1.8) |  |
| χ^2^_3_ | 2.4 | |  | 4.4 | |  | 3.7 | |  | 12.6* | |  |  |  |
| III. Combined preference |  |  |  |  |  |  |  |  |  |  |  |  |  | 34.8* |
| Very severe depression | 128 (29.2) | 2.0* (1.3, 3.1) |  | 87 (19.6) | 1.6* (1.0, 2.5) |  | 98 (23.7) | 2.0* (1.3, 3.0) |  | 67 (15.9) | 1.1 (0.7, 1.8) |  | 38 (11.6) |  |
| Severe depression | 143 (24.9) | 1.3 (0.9, 1.9) |  | 104 (19.0) | 1.2 (0.8, 1.7) |  | 139 (25.9) | 1.7* (1.1, 2.3) |  | 72 (14.3) | 0.8 (0.5, 1.1) |  | 71 (15.9) |  |
| Moderate depression | 248 (26.4) | 1.2 (0.9, 1.6) |  | 167 (17.7) | 1.0 (0.7, 1.3) |  | 170 (19.6) | 1.0 (0.7, 1.4) |  | 136 (17.0) | 0.8 (0.6, 1.1) |  | 136 (19.2) |  |
| Mild depression | 191 (21.4) | 1.0 (Ref) |  | 158 (18.1) | 1.0 (Ref) |  | 163 (19.5) | 1.0 (Ref) |  | 155 (21.9) | 1.0 (Ref) |  | 116 (19.1) |  |
| χ^2^_3_ | 11.3* | |  | 6.0 | |  | 15.8* | |  | 5.2 | |  |  |  |
|  |  |  |  |  |  |  |  |  |  |  |  |  |  |  |

*Significant at the .05 level, two-sided test.

^†^The distribution of depressive symptom severity was: n=413 with very severe depression, n=529 with severe depression, n=857 with moderate depression, n=788 with mild depression.

^‡^Based on multinomial logistic regression models with severity predicting preferences controlling for socio-demographics and geographic variables.

Note. Data are weighted, but reported n’s are unweighted.

| **Appendix Table 4. Associations of depression severity and patient treatment preferences with treatment setting (specialty mental health versus primary care) (n=2,582)**^†^ | | | | |
| --- | --- | --- | --- | --- |
|  | | | | |
|  |  |  | | **Specialty mental health vs. primary care setting**^†^ |
|  | **n (%)** |  | | **OR (95% CI)** |
| A. Depression severity |  |  | |  |
| Very severe | 413 (15.9) |  | | 1.1 (0.8, 1.4) |
| Severe | 529 (20.8) |  | | 1.4* (1.1, 1.8) |
| Moderate | 857 (33.7) |  | | 0.9 (0.5, 1.1) |
| Mild | 783 (29.6) |  | | 1.0 Ref |
| χ^2^_3_ |  |  | | 12.4* |
| B. Preference for medication treatment | |  | |  |
| Strongly positive | 1065 (36.7) |  | | 0.8 (0.3, 2.7) |
| Positive | 413 (14.9) |  | | 0.9 (0.3, 3.2) |
| Neutral | 430 (17.0) |  | | 0.9 (0.3, 3.0) |
| Negative | 353 (15.4) |  | | 1.9 (0.5, 7.2) |
| Strong negative | 321 (16.0) |  | | 1.0 (Ref) |
| χ^2^_4_ |  |  | | 5.6 |
| C. Preference for psychotherapy treatment | | |  |  |
| Strongly positive | 1312 (51.2) |  | | 1.9 (0.7, 5.0) |
| Positive | 657 (25.5) |  | | 1.5 (0.6, 4.1) |
| Neutral | 419 (16.2) |  | | 1.6 (0.6, 4.4) |
| Negative | 136 (5.1) |  | | 1.5 (0.5, 4.3) |
| Strongly negative | 58 (2.0) |  | | 1.0 (Ref) |
| χ^2^_4_ |  |  | | 4.6 |
| D. Preference for combined treatment | |  | |  |
| Strongly positive | 710 (25.1) |  | | 1.1 (0.3, 3.6) |
| Positive | 516 (18.4) |  | | 0.9 (0.3, 3.3) |
| Neutral | 570 (21.5) |  | | 1.0 (0.3, 3.4) |
| Negative | 425 (17.7) |  | | 0.6 (0.2, 2.2) |
| Strongly negative | 361 (17.3) |  | | 1.0 (Ref) |
| χ^2^_4_ |  |  | | 2.6 |
| χ^2^_15_ |  |  | | 34.1* |
|  |  |  | |  |

*Significant at the .05 level, two-sided test.

^†^Interaction models were also estimated that tested the significance of interactions between depression severity and preferences for medication (χ^2^_12_= 17.1, p=0.15), psychotherapy (χ^2^_12_= 8.6, p=0.74), and combined (χ^2^_12_= 14.1, p=0.30) treatments in predicting treatment sector.

Note. Data are weighted, but reported n’s are unweighted.

| **Appendix Table 5. Associations of patient treatment preferences, depression severity, and treatment setting with types of treatment received in fully adjusted models (n=2,582)**^†^ | | | | | | |
| --- | --- | --- | --- | --- | --- | --- |
|  | | | | | | |
|  | **Types of Treatment Received** | | | | | |
|  | **Medication**^‡^ |  | **Psychotherapy**^§^ | |  | **Combined**^‖^ |
|  | **OR (95% CI)** |  | | **OR (95% CI)** |  | **OR (95% CI)** |
| A. Preferences for the outcome treatment type |  |  | |  |  |  |
| Strongly positive | 18.2* (12.9, 25.7) |  | | 1.8 (1.0, 3.3) |  | - |
| Positive | 15.5* (10.6, 22.6) |  | | 1.7 (0.9, 3.2) |  | - |
| Neutral | 7.1* (4.9, 10.2) |  | | 1.4 (0.7, 2.6) |  | - |
| Negative | 4.5* (3.1, 6.5) |  | | 1.3 (0.6, 2.7) |  | - |
| Strongly negative | 1.0 (Ref) |  | | 1.0 (Ref) |  | - |
| χ^2^_4_ | 355.3* |  | | 8.3 |  |  |
| B. Joint additive preferences for medication and psychotherapy treatments |  |  | |  |  |  |
| Medication |  |  | |  |  |  |
| Positive | - |  | | - |  | 1.8* (1.4, 2.4) |
| Neutral | - |  | | - |  | 1.0 (Ref) |
| Negative | - |  | | - |  | 0.5* (0.4, 0.7) |
| χ^2^_2_ |  |  | |  |  | 111.0* |
| Psychotherapy |  |  | |  |  |  |
| Positive | - |  | | - |  | 1.1 (0.8, 1.4) |
| Neutral | - |  | | - |  | 1.0 (Ref) |
| Negative | - |  | | - |  | 0.7 (0.4, 1.1) |
| χ^2^_2_ |  |  | |  |  | 4.1 |
| C. Depression severity |  |  | |  |  |  |
| Very severe | 1.0 (0.8, 1.3) |  | | 1.3 (1.0, 1.8) |  | 1.4* (1.1, 1.9) |
| Severe | 1.2 (0.9, 1.5) |  | | 0.9 (0.7, 1.2) |  | 1.2 (0.9, 1.6) |
| Moderate | 1.1 (0.9, 1.4) |  | | 0.9 (0.7, 1.2) |  | 1.1 (0.9, 1.4) |
| Mild | 1.0 (Ref) |  | | 1.0 (Ref) |  | 1.0 (Ref) |
| χ^2^_3_ | 1.8 |  | | 5.8 |  | 6.4 |
| D. Treatment setting |  |  | |  |  |  |
| SPMH | 1.0 (Ref) |  | | 1.0 (Ref) |  | 1.0 (Ref) |
| PC with full-time PC-MHI | 1.2 (1.0, 1.5) |  | | 0.5* (0.4, 0.6) |  | 0.7* (0.5, 0.8) |
| PC without full-time PC-MHI | 1.6 (0.9, 2.7) |  | | 0.2* (0.1, 0.4) |  | 0.2* (0.1, 0.4) |
| χ^2^_2_ | 5.7 |  | | 65.3* |  | 26.1* |
|  |  |  | |  |  |  |

*Significant at the .05 level, two-sided test.

^†^Based on multivariate models controlling for socio-demographics, geographic variables, childhood adversities and clinical severity.

^‡^Interaction models were also estimated that tested the significance of interactions between preferences for medication and depression severity (χ^2^_12_= 19.9, p=0.07), treatment setting (χ^2^_8_= 7.6, p=0.48), socio-demographics (χ^2^_36_= 55.5, p=0.02), geographic predictors (χ^2^_32_= 37.6, p=0.23), other aspects of clinical severity (χ^2^_28_= 28.1, p=0.46), and childhood adversities (χ^2^_8_= 9.1, p=0.91) in predicting medication adherence. The only one of these that was significant, with socio-demographics, was no longer significant after correcting for the false discovery rate.

^§^Interaction models were also estimated that tested the significance of interactions between preferences for psychotherapy and depression severity (χ^2^_12_= 12.0, p=0.44), treatment setting (χ^2^_8_= 2.6, p=0.96), socio-demographics (χ^2^_36_= 25.7, p=0.90), geographic predictors (χ^2^_32_= 24.7, p=0.78), other aspects of clinical severity (χ^2^_28_= 27.6, p=0.49), and childhood adversities (χ^2^_8_= 10.0, p=0.87) in predicting psychotherapy adherence.

^‖^Interaction models were also estimated that tested the significance of interactions between preferences for medication and psychotherapy with depression severity (χ^2^_12_= 4.5, p=0.97), treatment setting (χ^2^_8_= 9.1, p=0.33), socio-demographics (χ^2^_36_= 38.8, p=0.34), geographic predictors (χ^2^_32_= 29.1, p=0.61), other aspects of clinical severity (χ^2^_28_= 25.1, p=0.62), and childhood adversities (χ^2^_8_= 10.9, p=0.82) in predicting combined treatment adherence.

Note. Data are weighted, but reported n’s are unweighted.

| **Appendix Table 6. Associations of patient treatment preferences, depression severity, and treatment setting in predicting treatment adherence among patients either prescribed medication, referred for psychotherapy, or both in fully adjusted models**^†^ | | | | | |
| --- | --- | --- | --- | --- | --- |
|  | | | | | |
|  |  | | | | |
|  | **Medication Adherence**  **(n=1,608)** |  | **Psychotherapy Adherence**  **(n=1,774)** |  | **Combined Adherence**  **(n=800)** |
|  | **Multivariate**^‡^ |  | **Multivariate**^§^ |  | **Multivariate**^‖^ |
|  | **OR (95% CI)** |  | **OR (95% CI)** |  | **OR (95% CI)** |
| A. Preferences for the outcome treatment type (i.e., medication or psychotherapy) |  |  |  |  |  |
| Strongly positive | 1.7 (0.8, 3.3) |  | 3.3* (1.4, 7.4) |  | - |
| Positive | 1.1 (0.6, 2.3) |  | 2.6* (1.1, 6.0) |  | - |
| Neutral | 1.2 (0.6, 2.4) |  | 1.6 (0.7, 3.8) |  | - |
| Negative | 0.8 (0.4, 1.8) |  | 1.2 (0.5, 3.1) |  | - |
| Strongly negative | 1.0 (Ref) |  | 1.0 (Ref) |  | - |
| χ^2^_4_ | 15.3* |  | 40.6* |  |  |
| B. Joint additive preferences for medication and psychotherapy treatments |  |  |  |  |  |
| 1.Medication |  |  |  |  |  |
| Positive | - |  | - |  | 1.7 (0.9, 3.3) |
| Neutral | - |  | - |  | 1.0 (Ref) |
| Negative | - |  | - |  | 1.1 (0.5, 2.5) |
| χ^2^_2_ |  |  |  |  | 4.2 |
| 2. Psychotherapy |  |  |  |  |  |
| Positive | - |  | - |  | 1.2 (0.6, 2.3) |
| Neutral | - |  | - |  | 1.0 (Ref) |
| Negative | - |  | - |  | 0.7 (0.2, 3.0) |
| χ^2^_2_ |  |  |  |  | 0.9 |
| C. Depression severity |  |  |  |  |  |
| Very severe | 0.8 (0.6, 1.2) |  | 1.2 (0.9, 1.6) |  | 0.8 (0.4, 1.6) |
| Severe | 0.8 (0.6, 1.2) |  | 1.1 (0.8, 1.4) |  | 1.1 (0.6, 1.9) |
| Moderate | 0.9 (0.7, 1.2) |  | 0.9 (0.7, 1.2) |  | 1.0 (0.6, 1.7) |
| Mild | 1.0 (Ref) |  | 1.0 (Ref) |  | 1.0 (Ref) |
| χ^2^_3_ | 1.2 |  | 3.3 |  | 0.7 |
| D. Treatment setting |  |  |  |  |  |
| SPMH | 1.0 (Ref) |  | 1.0 (Ref) |  | 1.0 (Ref) |
| PC with FT PC-MHI | 1.0 (0.7, 1.3) |  | 1.1 (0.9, 1.3) |  | 0.9 (0.6, 1.5) |
| PC without FT PC-MHI | 0.6 (0.3, 1.2) |  | 1.2 (0.6, 2.5) |  | 0.1 (0.0, 12.0) |
| χ^2^_2_ | 2.0 |  | 0.5 |  | 0.9 |
|  | | | | | |

*Significant at the .05 level, two-sided test.

^†^Based on multivariate models controlling for socio-demographics, geographic variables, childhood adversities and clinical severity.

^‡^Interaction models were also estimated that tested the significance of interactions between preferences for medication and depression severity (χ^2^_12_= 20.5, p=0.06), treatment setting (χ^2^_8_= 6.1, p=0.63), socio-demographics (χ^2^_36_= 27.4, p=0.50), geographic predictors (χ^2^_32_= 17.2, p=0.98), other aspects of clinical severity (χ^2^_28_= 27.4, p=0.61), and childhood adversities (χ^2^_8_= 13.9, p=0.61) in predicting medication adherence.

^§^Interaction models were also estimated that tested the significance of interactions between preferences for psychotherapy and depression severity (χ^2^_12_= 9.8, p=0.64), treatment setting (χ^2^_8_= 15.6, p=0.03), socio-demographics (χ^2^_36_= 41.0, p=0.26), geographic predictors (χ^2^_32_= 27.9, p=0.63), other aspects of clinical severity (χ^2^_28_= 24.5, p=0.65), and childhood adversities (χ^2^_8_= 15.3, p=0.50) in predicting psychotherapy adherence.

^‖^Interaction models were also estimated that tested the significance of interactions between preferences for medication and psychotherapy with depression severity (χ^2^_12_= 14.3, p=0.28), treatment setting (χ^2^_6_= 2.8, p=0.83), socio-demographics (χ^2^_36_= 29.6, p=0.76), geographic predictors (χ^2^_31_= 20.7, p=0.92), other aspects of clinical severity (χ^2^_28_= 16.4, p=0.96), and childhood adversities (χ^2^_15_= 4.8, p=0.99) in predicting combined treatment adherence.

Note. Data are weighted, but reported n’s are unweighted.

**Appendix Figure 1. Flow diagram of patients recruited into the study among those seen for incident depression as reported in the Veterans Health Administration electronic medical records from 12/2018 - 6/2020.**

Unique Patients
n=55,106

No Contact (n=38,106)

- *Maximum number of call attempts reached n=27,603*
- *Non-working contact n=6,828*
- *Moved, no forwarding n=3,675*

Contact Made
n=17,000

Refusals (n=10,702)

- *Refused n=10,659*
- *Rescinded n=43*

Consented
n=6,298

Incomplete Baseline (n=2,162)

- *Didn’t start n=1,688*
- *Didn’t finish n=446*
- *Didn’t complete ADM and psychotherapy preference questions n=28*

Not Eligible (n=1,554)

- *Suicidal n=84*
- *Depression not a presenting problem n=471*
- *Mania a presenting problem n=728*
- *Reported no depression severity n=271*

Analytic Sample
n=2,582

Consented and Complete
n=4,136

**APPENDIX REFERENCES**

1. **Centers for Disease Control and Prevention**. The International Classification of Diseases, Ninth Revision, Clinical Modification (ICD-9-CM). National Center for Health Statistics. 2013. https://www.cdc.gov/nchs/icd/icd9cm.htm. Accessed January 18, 2021.

2. **Centers for Disease Control and Prevention**. The International Classification of Diseases, Tenth Revision, Clinical Modification (ICD-10-CM). National Center for Health Statistics. 2019. https://www.cdc.gov/nchs/icd/icd10cm.htm. Accessed January 18, 2021.
